# Septic serum induces glucocorticoid resistance and modifies the expression of glucocorticoid isoforms receptors: a prospective cohort study and *in vitro *experimental assay

**DOI:** 10.1186/cc12774

**Published:** 2013-06-12

**Authors:** Julia Guerrero, Héctor A Gatica, Margarita Rodríguez, Roberto Estay, Irmgadt Annelise Goecke

**Affiliations:** 1 Intensive Care Unit, Clinical Hospital, University of Chile, Santos Dumont 999, Independencia, Santiago, Chile; 2 Physiology and Biophysics Disciplinary Program, ICBM, Faculty of Medicine, University of Chile, Independencia 1027, Independencia, Santiago, Chile; 3 Support Office for Clinical Investigation, Clinical Hospital, University of Chile, Santos Dumont 999, Independencia, Santiago, Chile; 4 Rheumatology Service, Internal Medicine Department, Clinical Hospital, University of Chile Santos Dumont 999, Independencia, Santiago, Chile

**Keywords:** corticosteroids, glucocorticoid receptor, glucocorticoid receptor beta, inflammation, sepsis, septic shock

## Abstract

**Background:**

A protective role for glucocorticoid therapy in animal models of sepsis was shown many decades ago. In human sepsis, there is new interest in glucocorticoid therapy at a physiological dose after reports of improved response to vasopressor drugs and decreased mortality in a selected group of patients. However, other reports have not confirmed these results. Cellular glucocorticoid resistance could explain a possible cause of that. To evaluate this hypothesis, we evaluated the expression of glucocorticoid receptor beta, the dominant negative isoform of glucocorticoid receptor, in peripheral mononuclear cells of septic patients and the effect of serum septic patients over glucocorticoid receptor expression and glucocorticoid sensitivity in immune cells culture.

**Methods:**

A prospective cohort study and an *in vitro *experimental study with matched controls were developed. Nine patients with septic shock and nine healthy controls were prospectively enrolled. Mononuclear cells and serum samples were obtained from the patients with sepsis on admission to the Intensive Care Unit and on the day of discharge from hospital, and from healthy volunteers matched by age and sex with the patients. Glucocorticoid receptor alpha and beta expression from patients and from immune cell lines cultured in the presence of serum from septic patients were studied by western blot. Glucocorticoid sensitivity was studied in control mononuclear cells cultured in the presence of serum from normal or septic patients. A statistical analysis was performed using a Mann-Whitney test for non-parametric data and analysis of variance for multiple comparison; *P *< 0.05 was considered significant.

**Results:**

The patients' glucocorticoid receptor beta expression was significantly higher on admission than on discharge, whereas the alpha receptor was not significantly different. *In vitro*, septic serum induced increased expression of both receptors in T and B cells in culture, with a greater effect on receptor beta than the control serum. Septic serum induced glucocorticoid resistance in control mononuclear cells.

**Conclusion:**

There is a transient increased expression of glucocorticoid receptor beta in mononuclear cells from septic patients. Serum from septic patients induces cell glucocorticoid resistance *in vitro*. Our findings support a possible cell glucocorticoid resistance in sepsis.

## Introduction

Sepsis is a leading cause of death in intensive care units (ICUs) around the world [1]. In spite of new developments in critical care and sepsis therapy, the mortality rates associated with severe sepsis and septic shock are still over 30% in most reports [1-5].

A protective role for glucocorticoid (GC) therapy in animal models of sepsis was shown many decades ago [6,7], leading to a proposal as early as 1940 for the use of GCs to treat patients with severe sepsis [8]. However this therapeutic strategy has had several dramatic shifts with time. The early approach of high dose GCs for sepsis therapy was abandoned when the potential benefits initially reported [9-11] could not be replicated, and higher mortality associated with secondary infections was suggested [12,13]. A renewed interest in GC therapy in sepsis, at what has been termed a physiological dose, was seen after reports of improved response to vasopressor drugs and decreased mortality in selected groups of patients who had an inadequate response to adrenocorticotropin hormone (ACTH) (defined as an increase in total plasma cortisol <9 μg/dL) [14,15]. However, in 2008, the CORTICUS study [16] did not confirm these results. This contradictory evidence still leaves much uncertainty about the real benefits of GC therapy in sepsis [17-19]. The most accepted conclusion from earlier studies was that positive GC effects in sepsis were seen only in a restricted group of patients. Until recently, these patients had been identified through a reduced ACTH response, which has been termed adrenal insufficiency or inadequate response to stress [14]. This selection method and its role as a prognostic factor has also been challenged [20-25]. Therefore it is possible that to demonstrate any benefits for GC therapy in sepsis, it will be necessary to define further to whom GC therapy should be prescribed.

It is possible that target cells in some patients with appropriate cortisol plasma levels do not fully respond to GCs, being less sensitive to a given GC concentration - a phenomenon called GC resistance [26-28]. These patients, if they exist, would not be identified by the ACTH response test but they could benefit from steroid therapy.

The actions of GCs are mediated by their receptor (GR), which acts as a ligand-dependent transcription factor [29-31]. Although the GR is the product of a single gene, several isoforms have been described [32,33]. The most studied are GR alpha (GRα), the classical receptor that mediates GC actions, and GR beta (GRβ), which can act as a dominant negative [34-37]. A change in the expression of the GR isoforms with greater GRβ expression has been proposed as a mechanism of GC resistance in chronic inflammatory conditions such as rheumatoid arthritis, ulcerative colitis and others [38-47]. Previous studies of the effect of sepsis on GR expression in humans and endotoxin-treated rats have shown apparently contradictory results in different cell types and tissues, leading the authors to suggest a cell-specific GR modulation of expression in sepsis [48-52]. However, some of the above studies have evaluated only the mRNA levels of the GR, which do not necessarily correlate with the receptor protein expression [47], while others have evaluated the GR protein expression using antibodies that do not discriminate between GRα and GRβ or by GC binding assays, which evaluate only GRα [51].

It is known that normal mononuclear cells (lymphocytes and monocytes) are sensitive to the actions of GCs. These hormones can influence the T helper 1 and 2 cells balance and modulate cytokine production, which subsequently influences the inflammatory response in sepsis. It is therefore important to know if there is a change in GC receptor expression and/or sensitivity in mononuclear cells in sepsis.

It has been demonstrated that inflammatory cytokines *in vitro *can preferentially increase the expression of GRβ in lymphocytic cell lines and induce GC resistance [51]. It is possible that proinflammatory cytokines or other factors present in the serum of septic patients may induce a greater expression of GRβ leading to a GC-resistant state. This response could be an appropriate feedback loop in infectious diseases but it also might lead to an unbalanced inflammatory response, which contributes to the morbidity of sepsis.

This hypothesis must be evaluated because, at least in theory, these patients could benefit from GC supplementation to overcome their lower cell GC sensitivity, even in cases where an adequate basal cortisol level or adequate ACTH response can be documented.

The aims of this study were:

1. To evaluate whether there is an overexpression of the dominant-negative GCβ in peripheral mononuclear cells (PBMCs) of septic patients.

2. To assess whether factors present in the serum of septic patients modulate the *in vitro *expression of GRs and/or cell GC sensitivity.

## Methods

### Selection of patients and controls

Patients 18 years or older, admitted to the ICU of University of Chile Clinical Hospital (November 2004 to December 2005) were prospectively enrolled in this study. The Institutional Ethics Board of the University of Chile Clinical Hospital approved the study and informed consent was obtained from all patients or relatives. The inclusion criteria for this study were:

1. Persistent arterial hypotension - systolic arterial pressure below 90 mmHg, mean arterial pressure <60 mmHg or a reduction in systolic blood pressure of >40 mmHg from baseline despite adequate volume resuscitation in the absence of other causes of hypotension;

2. Presence of at least one organ dysfunction as defined by Marshall or by the definition used for the Sequential Organ Failure Assessment score;

3. Infection focus site suspected or documented;

4. Elements of systemic inflammatory response - body temperature >38°C or <36°C, heart rate >90 beats/min, respiratory rate >20 breaths/min, white blood count >12,000 μL^-1^, <4,000 μL^-1 ^or >10% immature forms.

Exclusion criteria were:

1. GC supplementation prior to admission to ICU;

2. Hypothalamic-pituitary-adrenal axis dysfunction;

3. GC therapy within 6 months prior to admission to the ICU;

4. HIV or advanced cancer disease;

5. Chronic inflammatory disease;

6. Pregnancy.

Demographic data, severity score of septic event, focus of infection, identified microbiological agent and outcome were consigned.

Healthy volunteers (*n *= 9) with no history of inflammatory or autoimmune diseases, matched by age and sex with patients, were included in the study after informed consent was given. Exclusion criteria were the same as for the patient group.

### Hypothalamic-pituitary-adrenal axis evaluation

On ICU admission, the total cortisol serum and adrenal reserve (Synacthen test) were evaluated for all patients. Briefly, 250 μg of cosyntropin (Novartis, Basel, Switzerland) was administered intravenously. Serum samples were obtained immediately before cosyntropin administration and 30 minutes and 60 minutes after. The samples were stored at -80°C for cortisol level analysis. The hormone level was quantified by a commercial kit (Immunlite Cortisol^®^, Diagnostic Products Corporation, Los Angeles, CA, USA).

### Peripheral blood mononuclear cell preparation

Within 24 hours of admission, before GC supplementation, and on the third day in the ICU, 30 ml of venous blood was obtained from control individuals and from all patients. Another blood sample was obtained from patients that survived the septic event on the day of hospital discharge. Peripheral blood mononuclear cells were isolated immediately by density gradient centrifugation (Ficoll-Paque™ Plus, GE Healthcare.

### Human cell lines

Human immune cell lines (CEM, Raji and K562 cells) were cultured in Roswell Park Memorial Institute 1640 medium enriched with human septic or normal serum, 10% final concentration at 37°C in a 5% CO_2 _humidified atmosphere.

### Western blot

The expression of the cells' GR isoforms was evaluated by western blotting using specific antibodies for GRα (GR P-20: sc1002; Santa Cruz Biotechnology Inc., Santa Cruz, CA, USA) and GRβ (PA3-514; Affinity BioReagents, Golden, CO, USA), as described previously [47]. Briefly, 40 μg of total protein extract was loaded on 10% SDS-PAGE gels. The resolved proteins were transferred onto nitrocellulose membranes (30 mV, overnight at 4°C). The membranes were blocked with 10% non-fat milk and incubated at room temperature with the anti-GRα antibody (1:200; GR (8P-20): sc-1002, Santa Cruz Biotechnology, Inc.) or anti-GRβ antibody (1:500; PA3-514 Affinity Bioreagents, Inc., now called Thermo Scientific Pierce Antibodies for 16 hours and 36 hours, respectively, and for 1 hour with mouse monoclonal anti-actin (1:40,000) antibody (Santa Cruz Biotechnology, Inc.). Secondary biotinylated polyclonal antibodies against rabbit or mouse IgG (DAKO) were used for GR and actin detection, respectively. Immunoreactivity was detected by the enhanced chemiluminescence method (Amersham Bioscience, Amersham, UK). The densitometric analysis of the bands was performed using the Scion Image beta 4.02 win software.

### Glucocorticoid sensitivity assay

GC sensitivity was evaluated by measuring the dexamethasone inhibition of the lipopolysaccharide (LPS)-induced TNFα released. PBMCs obtained from healthy human volunteers were cultured in the presence of human septic serum or human normal serum for 48 hours. During the last 6 hours of culture, LPS 10 μg/ml (*Escherichia coli *0111:B4) with or without dexamethasone (10^-7 ^M) was added. The supernatants were obtained and analyzed for TNFα levels by ELISA assay (Biotrak Easy ELISA, Biotrak, Amersham, UK).

### Statistical analysis

For multiple comparisons, results were analyzed by analysis of variance. For comparisons between two values, the nonparametric Mann-Whitney test was used. A *P *value < 0.05 was considered significant. Data are expressed as medians (range).

## Results

### Patients' characteristics and hypothalamic-pituitary-adrenal axis evaluation

Three men and six women with septic shock, median age 63 years old (range: 33 to 75 years) were included in this study (Table 1). The median Acute Physiology and Chronic Health Evaluation II score at admission was 27 (range: 18 to 35). The most common septic sources were abdomen (55.6%) and lungs (33.3%). The microorganism was identified in 55.6% (five) of the patients. A negative Gram stain was obtained in three of these and a positive Gram stain was obtained in the remaining two. The median cortisol level at ICU admission was 26.43 μg/dL (range: 13.4 μg/dL to 61.5 μg/dL); the median cortisol levels 30 and 60 minutes after the administration of cosyntropin were 34.7 μg/dL (range: 22.3 μg/dL to 61.5 μg/dL) and 35.8 μg/dL (range: 22.5 μg/dL to 61.5 μg/dL), respectively. There was an increase in total plasma cortisol >9 μg/dL in 77.8% of the patients after cosyntropin administration. Patients received GC supplementation according to international recommendation [5] (Table 1).

**Table 1:** Patients' clinical characteristics

	All	Survivor	Non-survivor
Age, years	63 (33 to 75)	61.5 (33 to 75)	56.8 (37 to 73)
			
Gender	3 male/ 6 female	2 male 3 female	1 male 3 female
			
Infectious site:			
abdominal, n	5 (55.5%)	2	3
lung, n	3 (33.3%)	1	2
urinary, n	1 (11.1%)	1	0
			
Infectious agent demonstrated, n:	5 (55.6%)	3	2
gram positive, n	2	1	1
gram negative, n	3	2	1
			
APACHE II	27 (18 to 35)	25 (23 to 32)	27 (18 to 35)
			
Plasma cortisol level, μg/dL:			
basal	26.43 (13.4 to 61.5)	23.7 (26.85)	29.7 (34.78)
30 minutes after Synacthen^®^	34.7 (22.3 to 61.5)	35.5 (39.4)	31.6 (36.75)
60 minutes after Synacthen^®^	35.8 (22.5 to 61.5)	38.35 (40.98)	32.1 (37.05)
Alive/dead, n		5 (55.5%)	4 (44.4%)

APACHE II, Acute Physiology and Chronic Health Evaluation II.

### Glucocorticoid receptor expression in the peripheral blood mononuclear cells of septic patients

To evaluate whether sepsis is associated with changes in PBMC GR isoform expression, western blotting was used to evaluate the GRα and GRβ cells in PBMCs from septic patients on ICU admission and on the hospital discharge day, which was considered as the control situation (free of sepsis) for each patient.

**Figure 1:**
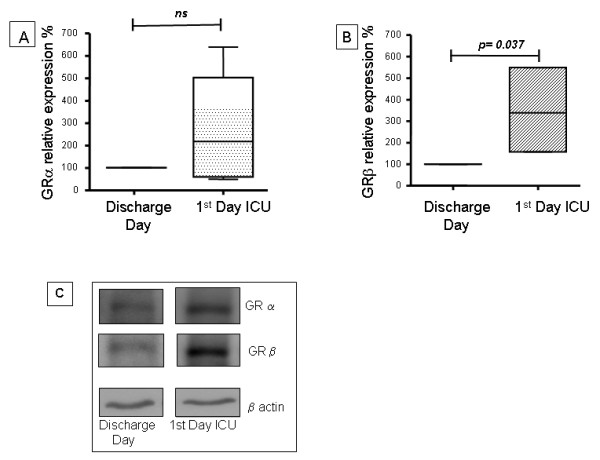
**Western blot analysis of glucocorticoid receptor α and β isoforms of peripheral blood mononuclear cells from septic patients**. GRα and GRβ cell expression were evaluated by western blotting in peripheral blood mononuclear cells from septic patients on ICU admission (first day in the ICU) and on the day of hospital discharge, which was considered as the control situation (free of sepsis). The quantification of the western blot analysis of **(A) **GRα and **(B) **GRβ is shown. The values were normalized to β actin expression and are expressed as percentage values of the control (GR expression on the day of hospital discharge). Data are presented as median value, 25% to 75% (box) and minimum-maximum (vertical line). **(C) **Representative western blot analysis of GRαGRβ and β actin are shown. GR, glucocorticoid receptor; ICU, intensive care unit.

**Figure 2:**
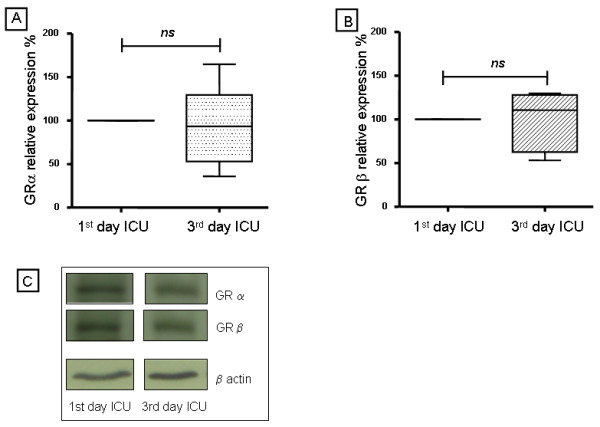
**Western blot analysis of glucocorticoid receptor α and β isoforms of peripheral blood mononuclear cells from septic patients on first and third day in the intensive care unit**. The GRα and GRβ cell expressions were evaluated by western blotting in peripheral blood mononuclear cells from septic patients on the first and third day in ICU. The quantification of the western blot analysis of **(A) **GRα and **(B) **GRβ are shown. The values were normalized to β actin expression and are expressed as percentage values of the GR expression on the first day in ICU. Data are presented as median value, 25% to 75% (box) and minimum-maximum (vertical line). *ns *= not statistically significant. **(C) **Representative western blot analysis of GC receptors αβ and β actin are shown. GR, glucocorticoid receptor; ICU, intensive care unit.

Our results showed that all patients had significantly increased GRβ expression in PBMCs obtained at ICU admission compared to their expression at the time of hospital discharge (*n *= 5, *P *= 0.037), whereas the expression of GRα had not significantly changed (Figure 1). The level of expression of both GR isoforms observed on the first day in ICU was not significantly different by the third day (Figure 2).

**Figure 3:**
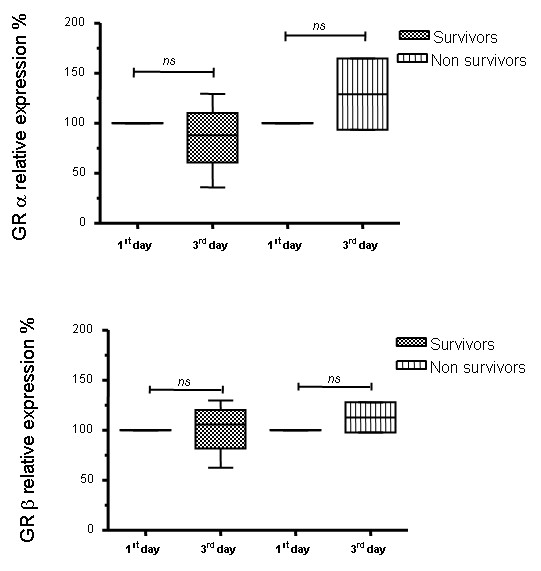
**Glucocorticoid receptor isoform expression on the first and third day in the intensive care unit for the survivors and non-survivors of sepsis**. GRα and β cell expression were evaluated on the first and third days in the ICU by western blotting of peripheral blood mononuclear cells from septic patients who survived or did not survive the septic event. The quantification of the western blot analysis of **(A) **GRα and **(B) **GRβ are shown. The values were normalized to β actin expression and are expressed as percentage values of the GR expression on the first day in the ICU. Data are presented as median value, 25% to 75% (box) and minimum-maximum (vertical line). *ns *= not statistically significant. **(C) **Representative western blot analysis of GRα, β and β actin are shown. GR, glucocorticoid receptor; ICU, intensive care unit.

We also separately evaluated the change in GR isoform expression between these dates for survivors and non-survivors among patients (mortality on 28th day of admission). Figure 3 shows that even though none of the values reached a statistically significant difference, there was a trend for an increased expression of GRα in non-survivors at third day in comparison with the first day in UCI. This trend was not observed in the survivors.

### Effect of septic serum on glucocorticoid receptor isoform expression in different cell lines

It is possible that cytokines or other factors present in the serum of septic patients induce changes in the cell expression of GR isotypes. To evaluate this hypothesis, we cultured three different human immune cell lines (CEM, Raji and K562) in the presence of serum from septic patients or serum obtained from the control group at 10% final concentration in the culture medium. After 48 hours in culture, the GR protein expression was analyzed by western blotting with specific antibodies for both GR isoforms.

**Figure 4:**
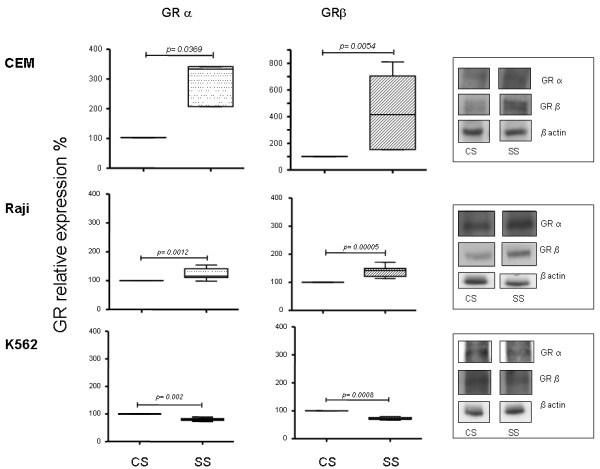
**Effect of human septic serum on glucocorticoid receptor isoform expression in different cell lines**. CEM, Raji and K562 cell lines were cultured in the presence of the patients' septic serum and serum obtained from the control group at 10% final concentration in the culture medium. After 48 h in culture, the GR protein expression was analyzed by western blotting with specific antibodies for GRα and β isoforms. The quantification of the western blot analysis of **(A) **GRα and **(B) **GRβ are shown (*n *= 9). The values were normalized to β actin expression and are expressed as percentage values of the control (GR cell expression cultured in the presence of control serum). Data are presented as median value, 25% to 75% (box) and minimum-maximum (vertical line). Representative western blot analysis of GRα, β and β actin are shown for each cell line. CS, control serum; GR, glucocorticoid receptor; SS, septic serum.

Figure 4 shows that septic serum induced significantly higher expression of both GRα and β than serum from healthy participants in CEM cells, a human T lymphoid cell line. The increased expression was greater in GRβ than GRα. A similar but less potent effect was observed for a human B lymphoid cell line, Raji. However, septic serum had an inverse effect on K562 cells, a human cell line derivate of chronic myelogenous leukemia. The expression of both GR isoforms in K562 cells cultured in the presence of septic serum was approximately 20% less than in cells cultured in the presence of control serum (Figure 4).

### Effect of septic serum on glucocorticoid cell sensitivity

**Figure 5:**
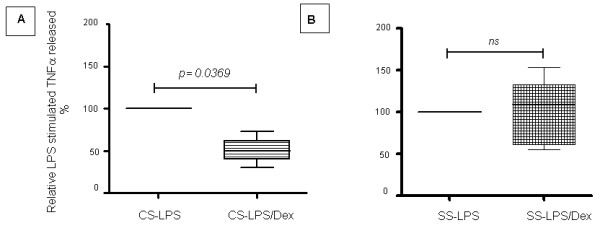
**Effect of septic serum on dexamethasone inhibition of lipopolysaccharide-induced TNFα release in normal human peripheral blood mononuclear cells**. The *in vitro *effect of septic serum on glucocorticoid sensitivity was evaluated in a dexamethasone cell sensitivity assay in control peripheral blood mononuclear cells in culture. Peripheral blood mononuclear cells from healthy control participants were cultured in the presence of pooled human septic serum taken from the patients on admission to the intensive care unit or pooled serum from the controls. After 48 h in culture, LPS or LPS plus dexamethasone 10^-7 ^M was added to the medium. The TNFα released in response to LPS stimuli in the presence and absence of dexamethasone was analyzed by ELISA. The values are expressed as a percentage of TNFα released from cells cultured in the presence of LPS without dexamethasone. *n *= 5. CS, control serum; Dex, dexamethasone; LPS, lipopolysaccharide; SS, septic serum, TNFα, tumor necrosis factor alpha.

Finally, to assess whether septic serum not only modifies GR isoform protein expression but also induces a change in GC cell sensitivity, we evaluated the effect of septic serum versus control serum on a dexamethasone cell sensitivity assay in control PBMCs in culture. These cells were cultured in the presence of pooled human septic serum taken from the patients on admission to ICU or pooled serum from the controls. After 48 hours in culture, LPS or LPS plus dexamethasone was added to the medium. Figure 5A shows that dexamethasone inhibited the LPS-induced TNFα release by PBMC cultured in the presence of normal serum. However, when cells were cultured in septic serum, dexamethasone lost its inhibitory effect (Figure 5B). The same experiment was also performed using pooled human septic serum from both surviving and non-surviving patients (data not shown), which resulted in a complete loss of dexamethasone inhibitory effect in both groups.

## Discussion

The role of GC therapy in septic patients is still controversial [19,53-55]. Dose, duration of treatment and selection of patients influences the benefit of the therapy [19,54]. Insufficient adrenal GC secretion and peripheral GC resistance are factors likely to influence the response of patients to corticosteroids [26-28]. GC actions are mediated through their cellular receptors. Because a dominant negative effect of GRβ was reported, a potential role for this receptor in GC-resistant states has been proposed [39-41,45]. Therefore our study evaluated the expression of GRα and β in septic patients. The expression of GR can have high inter-individual variability [52], so we chose to evaluate the effect of sepsis on the expression of the GR within the same individual. As it is impossible to know in advance who will develop sepsis, we compared the mononuclear GR expression in a septic state (admission to ICU) with the same patient's GR expression at discharge from hospital (clinical recovery). This strategy also allowed us to evaluate if the potential change in expression induced by sepsis is transient or not. However, only the survivors from sepsis could be studied (*n *= 5).

Our data showed no difference in the expression of GRα in PBMC in sepsis (Figure 1). However it is important to stress that we studied a limited number of septic patients (nine) and only the survivors (five) were included for this analysis. It is possible in a small sample like ours that a difference between groups can not be demonstrated, especially when there is high variability in inter-individual responses. However, even with this limited number of patients, our results showed that, in sepsis, there was a significant transient increased expression of GRβ in PBMC (Figure 1). Ledderose *et al. *[52] also found an increase of GRβ mRNA expression in T cells from septic patients compared to stimulated or non-stimulated T-cells of healthy donors. That study also had very high inter-individual variability with many overlapping results between groups [19]. Our results showed that all patients had a higher expression of GRβ on the ICU admission day.

In sepsis, there is first a severe inflammatory reaction, followed by an anti-inflammatory phase as a compensatory response, corresponding in general to the third to fifth days of sepsis evolution. Both stages are characterized by different cytokines (TNFα, IL6 and IL1 for the inflammatory stage, and IL10 and TGFβ for the anti-inflammatory stage), with potentially different effects on GR expression. We therefore evaluated if there was a change in GR expression by the third day in ICU. We showed that the levels of expression of both GRα and GRβ observed on ICU admission day were maintained on the third day in the ICU (Figure 2). To study further whether a change in expression of GR isoforms was associated with 28-day mortality rate, we evaluated the changes of GR expression between the first and third days in the individuals who survived versus those who did not survive the septic event. These results were not significantly different (Figure 3). However, it may be of interest that a tendency for an increased expression of GRα on the third day was observed only in the non-survivor group (Figure 3).

It has previously been shown that pro-inflammatory cytokines such as IL1 and TNFα increase the expression of GRα and GRβ in lymphocytic cell lines *in vitro*, with a stronger effect on the expression of the β isoform [52]. The serum of septic patients has high levels of proinflammatory cytokines, as well as other factors such as GCs, which could at least in part influence the expression of GR. To assess whether soluble factors present in the serum of septic patients induce changes in the cellular expression of GR, we tested the effect of serum taken both from the patients on admission to the ICU and from the control group of healthy individuals, matched by age and sex to the patients, on the expression of GRα and β in cells in culture. Because the PBMC are primarily T lymphocytes but also B-lymphocytes and monocytes, we evaluated the effect of septic serum on T (CEM) and B (Raji) lymphoid cell lines and in a myeloid cell line (K562). Our results showed that, compared to control serum, the septic serum induced an increased expression of both GR isoforms in T and B lymphoid cells, with a stronger effect in the expression of GRβ (Figure 4). This result is similar to the *in vitro *effect of proinflammatory cytokines on GR [51] and is also similar to the results obtained from PBMC in the same septic patients (Figure 1), supporting a role for factors present in serum during sepsis in the induction of GRβ expression. However, this same serum induced the contrary effect on K562 cells (Figure 4).

We have insufficient information to know whether these results reflect a specific regulatory effect of GCs in different cell types with particular roles in the immune response or if they are secondary to an abnormal behavior of different tumor cell lines. However, we have shown that septic serum can regulate the expression of GR cells in culture. Because the dominant negative effect of GRβ has been proved *in vitro *only when it has been overexpressed five times or more than GRα, the increased expression of GRβ in itself does not necessarily imply a cell GC-resistant state [34]. We went on to investigate whether septic serum is also able to impair the cell GC sensitivity in cells in culture. Figure 5A shows that dexamethasone significantly inhibits the LPS-induced TNFα secretion from PBMC cultured in the presence of pooled serum from control participants. However, when these cells were cultured in the presence of pooled septic serum, dexamethasone lost its inhibitory effect (Figure 5B). The concentration of dexamethasone used in this assay is greater than the equivalent reported free plasma cortisol levels in septic shock patients treated with low-dose hydrocortisone therapy [56]. Therefore, our results show that septic serum not only influences the expression of cell GR in culture, but can also induce a GC-resistant state.

Finally, to explore further if the serum from patients who survived the septic event had a different effect on the expression of GR and/or GC sensitivity of cells in culture compared to the serum of non-survivors, we tested the effect of the pooled serum obtained on admission to the ICU from both groups of patients on GR expression and on cell GC sensitivity, as described previously. Our results showed that the serum from both survivors and non-survivors induced the same effects in GR expression and in the GC sensitivity test as the pooled serum from all septic patients studied together (data not shown). Further studies with higher number of patients are required to corroborate this data due to the limitations of the small sample evaluated.

## Conclusions

We found a transient increased expression of the dominant negative GRβ in PBMC from septic patients. We also demonstrated that serum from septic patients influences the expression of GR transcriptional isoforms α and β in cell lines in culture, increasing the dominant negative GRβ in T and B cell lines. Septic serum also induced a GC-resistant state in PBMC from normal participants *in vitro*. Therefore our results support the presence of a peripheral GC-resistant state in septic patients associated with a higher expression of GRβ which could be caused, at least in part, by agents present in the serum of septic patients. The small sample of patients and controls (*n *= 9) included in this work means that further study is required to corroborate our data and to evaluate whether this peripheral GC resistance could be a factor that influences the response to corticosteroid treatment in septic patients.

## Key messages

• GC treatment in sepsis and septic shock is still controversial.

• A GC-resistant state associated with sepsis could be important to define who could benefit from GC treatment.

• This study suggests there is a transient increased expression of GRβ in mononuclear cells from septic patients. We also showed that septic serum induces cell GC resistance *in vitro*.

• Further study is necessary to evaluate if a higher expression of GRβ in septic patients correlates with a better outcome with GC therapy.

## Abbreviations

ACTH: adrenocorticotropin hormone; ELISA: enzyme-linked immunosorbent assay; GC: glucocorticoid; GR: glucocorticoid receptor; GRα: glucocorticoid receptor alpha; GRβ: glucocorticoid receptor beta; ICU: intensive care unit; Ig: immunoglobulin; LPS: lipopolysaccharide; PBMC: peripheral blood mononuclear cell; TGF: transforming growth factor; TNFα: tumor necrosis factor alpha.

## Competing interests

The authors declare that they have no competing interests.

## Authors' contributions

JG and IAG participated in the design of study, results' analysis, drew the diagrams and contributed to the manuscript. JG carried out clinical activity, molecular laboratory techniques and cellular cultures. MR and RE carried out glucocorticoid cell sensitivity experiments. HG carried out the statistical analysis. All authors read and approved the final manuscript version.
